# Dynamic Remodeling of Dendritic Arbors in GABAergic Interneurons of Adult Visual Cortex

**DOI:** 10.1371/journal.pbio.0040029

**Published:** 2005-12-27

**Authors:** Wei-Chung Allen Lee, Hayden Huang, Guoping Feng, Joshua R Sanes, Emery N Brown, Peter T So, Elly Nedivi

**Affiliations:** 1The Picower Institute for Learning and Memory, Massachusetts Institute of Technology, Cambridge, Massachusetts, United States of America; **2**Department of Brain and Cognitive Sciences, Massachusetts Institute of Technology, Cambridge, Massachusetts, United States of America; **3**Department of Mechanical Engineering and Division of Biological Engineering, Massachusetts Institute of Technology, Cambridge, Massachusetts, United States of America; **4**Department of Neurobiology, Duke University Medical Center, Durham, North Carolina, United States of America; **5**Department of Molecular and Cellular Biology, Harvard University, Cambridge, Massachusetts, United States of America; **6**MIT-Harvard Division of Health Science and Technology, Massachusetts Institute of Technology, Cambridge, Massachusetts, United States of America; **7**Neuroscience Statistics Research Laboratory, Department of Anaesthesia and Critical Care, Massachusetts General Hospital, Boston, United States of America; **8**Department of Biology, Massachusetts Institute of Technology, Cambridge, Massachusetts, United States of America; Howard Hughes Medical InstituteUnited States of America

## Abstract

Despite decades of evidence for functional plasticity in the adult brain, the role of structural plasticity in its manifestation remains unclear. To examine the extent of neuronal remodeling that occurs in the brain on a day-to-day basis, we used a multiphoton-based microscopy system for chronic in vivo imaging and reconstruction of entire neurons in the superficial layers of the rodent cerebral cortex. Here we show the first unambiguous evidence (to our knowledge) of dendrite growth and remodeling in adult neurons. Over a period of months, neurons could be seen extending and retracting existing branches, and in rare cases adding new branch tips. Neurons exhibiting dynamic arbor rearrangements were GABA-positive non-pyramidal interneurons, while pyramidal cells remained stable. These results are consistent with the idea that dendritic structural remodeling is a substrate for adult plasticity and they suggest that circuit rearrangement in the adult cortex is restricted by cell type–specific rules.

## Abstract 2

Chronic in vivo imaging of fluorescent-labeled neurons in adult mice reveals extension and retraction of dendrites in GABAergic non-pyramidal interneurons of the cerebral cortex.

## Introduction

Hubel and Wiesel's groundbreaking work in the 1960s and 1970s defined a critical period in brain development when manipulating visual inputs causes dramatic functional and structural changes in layer 4 of the primary visual cortex [1–3]. Since their finding that large-scale rearrangement of thalamic afferents in the visual cortex is restricted to a developmental critical period, the adult brain has been considered relatively hard-wired and limited in its capacity for structural change. Functional reorganization of primary sensory maps in the adult brain (reviewed in [4]), even across long distances [5], was explained as unmasking of existing connections and was not considered to require outright growth [6]. Although there are indications that the adult cortex is capable of anatomical change in response to peripheral manipulation, particularly in the superficial layers [7,8], the scale of change is small compared with the critical period and is difficult to detect against the general variance in the size and shape of cortical neurons. Moreover, such changes are seen only in response to external perturbation, leaving it unclear whether arbor remodeling normally occurs in the adult cortex on a day-to-day basis, and to what extent.

With the advent of new technologies to time-lapse image neuronal morphology in vivo [9], the issue is now being revisited. Repeated in vivo imaging of apical dendrites extending from layer 5 pyramidal neurons into the superficial layers has been used to investigate dendritic spine dynamics in both the somatosensory and the visual cortex [10–13]. Less attention has been paid to potential changes in the overall structure of dendritic arbors. In fact, it has been suggested that little if any structural plasticity occurs in the apical dendrites of layer 5 pyramidal neurons in the adult somatosensory cortex [10], or in the apical dendrites of mitral and tufted cells in the adult olfactory bulb [14]. No study, to our knowledge, has directly addressed the potential for structural dynamics in a cross-section of neurons that reflects the diversity of neocortical cell types. More specifically, the non-pyramidal neurons of the neocortex have yet to be the focus of investigation by in vivo imaging studies, despite their important role in adult cortical plasticity and in reorganization of cortical maps [15,16].

Here we investigate the dendritic arbor dynamics of pyramidal and non-pyramidal neurons in the superficial layers of the adult visual cortex in vivo. Our results show that while dendritic branches of pyramidal cells remain stable, non-pyramidal interneurons in these layers are dynamic, exhibiting a range of structural changes on a week-to-week basis.

## Results

### Repeated In Vivo Imaging of Cortical Neurons

**Figure 1:**
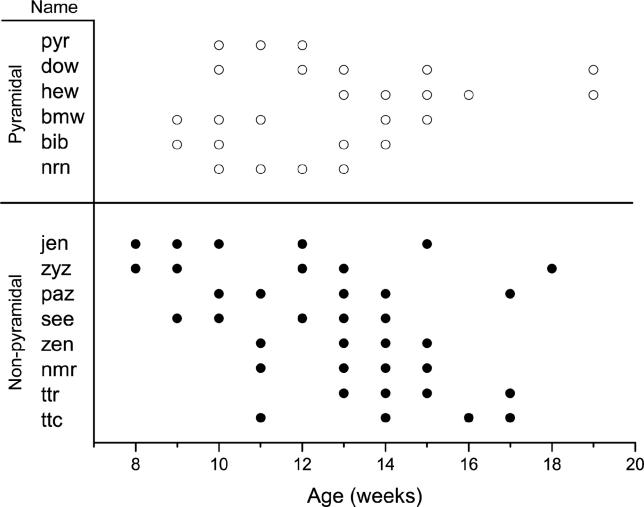
Summary of Imaging Sessions Displayed by Age Each neuron was named by an arbitrary three-letter code. Empty circles represent pyramidal neuron-imaging sessions, and filled circles represent non-pyramidal neuron-imaging sessions.

Studies suggest that intracortical connections in the supragranular layers of the neocortex, and in particular axonal sprouting, may be a locus of adult structural plasticity [7]. To directly test the prediction that neurons in layer 2/3 are capable of structural change and to assess the extent of dendritic structural dynamics in the adult cortex, we chronically imaged neuronal morphology in the intact rodent brain. To allow long-term visualization of neuronal structure in vivo, cranial windows were bilaterally implanted over the visual cortices of *thy1*-GFP-S mice [17] at 4–6 wk of age. These mice express green fluorescent protein (GFP) in a sparse pseudo-random subset of neocortical neurons. Imaging began at least 2 wk after surgery to allow for recovery and optical clarification of the cranial windows. Brains were screened for optically accessible GFP-positive neurons using wide-field fluorescence, and neuronal location was noted using local landmarks in the brain's surface vasculature. Individual GFP-labeled neurons in layer 2/3 of the visual cortex of anesthetized adult mice were then time-lapse imaged using a custom-made two-photon microscope [18]. To include as many neuronal branch tips as possible within the imaging volume, nine slightly overlapping volumes were imaged in a 3 × 3 array through *z–x–y* translation of an automated motorized stage. Individual image planes were stitched together to create a montage of adjoining *x–y* sections for a given depth from the pial surface. In an attempt to provide a comprehensive view of adult structural plasticity, data collection was initially not restricted to any particular cell type. Six pyramidal cells and eight non-pyramidal cells from 13 animals were time-lapse imaged for 3–10 wk (Figure 1), and four-dimensional morphometric analysis was carried out by quantitative comparison of dendritic branch tip length (BTL) as a function of time. Branch tips that were not imaged clearly for multiple imaging sessions or whose termination was unclear were excluded from analysis.

### Pyramidal Cells Are Stable Over Time

**Figure 2:**
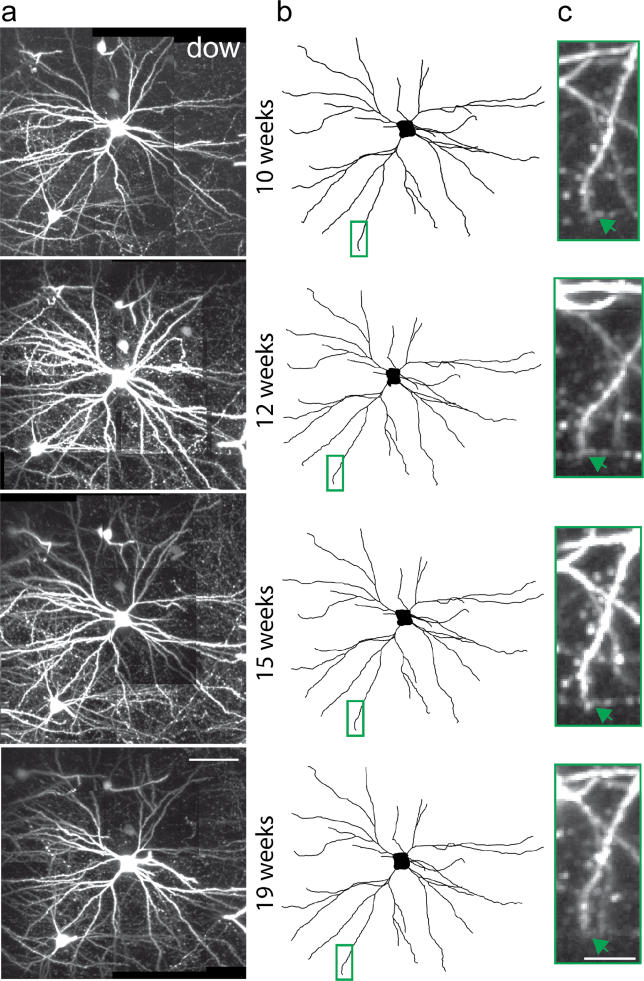
Dendritic Arbors of Pyramidal Neurons Are Stable (A) MZPs near the cell body of the pyramidal cell “dow” acquired over 9 wk. (B) Two-dimensional projections of three-dimensional skeletal reconstructions of “dow.” (C) High-magnification view of branch tip (green arrow) in region outlined by green box in (B). Scale bars: (A and B), 50 μm; (C), 10 μm.

Imaged pyramidal cells exhibited typical small pyramidal morphologies with a spiny apical dendrite and a skirt of spiny basal dendrites emanating from the lower half of a pyramid-shaped cell body. An example is shown in Figure 2 and in Video S1. The cell body of this pyramidal neuron, “dow,” was located 180 μm below the pial surface. It had a total of 57 dendritic branch tips on its apical dendrite and five primary basal dendrites. We monitored 28 of the 57 branch tips over 9 wk. Examination of individual branch tips revealed no overt sign of structural change (Figure 2A–2C). Similar examination of all cells in the pyramidal population did not identify any change in apical and basal dendritic branches. These data suggest that under normal conditions the dendritic branches of layer 2/3 pyramidal neurons in the visual cortex are relatively stable in the adult, and are consistent with previous studies reporting dendritic branch stability in other cortical areas [10,14].

### Dynamic Remodeling of Non-Pyramidal Neurons

**Figure 3:**
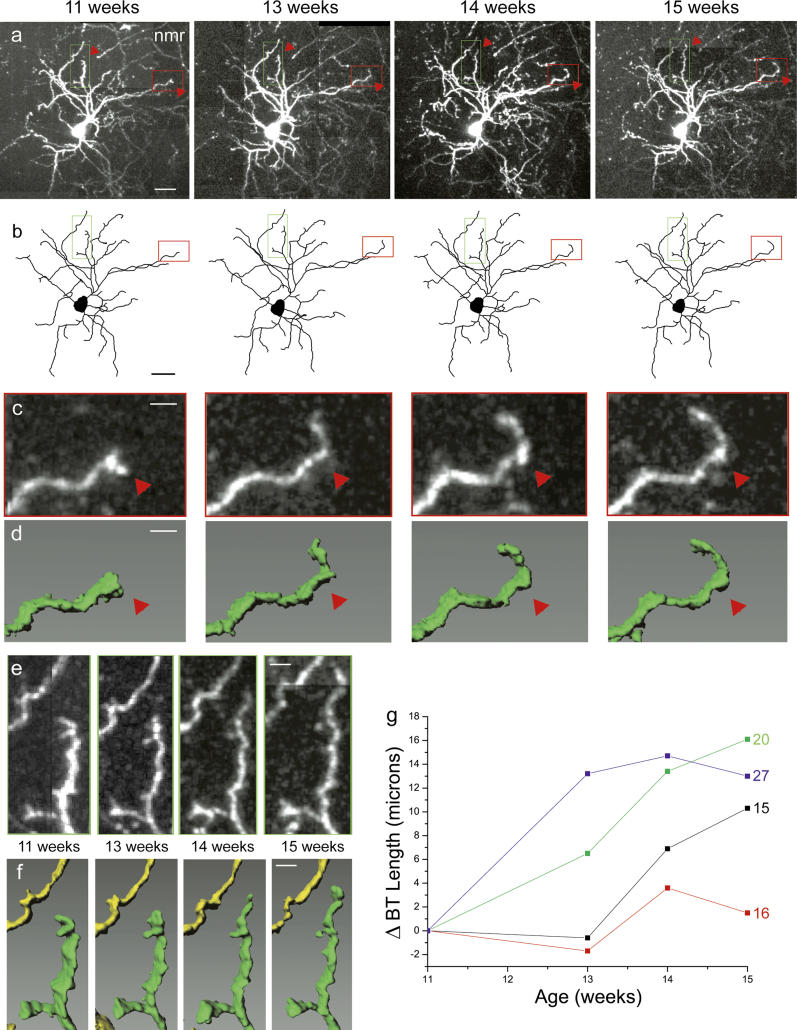
Dendritic Growth in Multiple Branches of a Non-Pyramidal Neuron (A) MZPs near the cell body of the (∼118-μm deep) non-pyramidal cell “nmr” acquired over 4 wk. Two examples of dendritic branch growth are indicated by red arrowheads. (B) Two-dimensional projections of three-dimensional skeletal reconstructions of the non-pyramidal neuron “nmr.” (C) High-magnification view of one growing branch tip (#20) (red box in [A and B]). Red arrowhead marks the approximate distal end of the branch tip at 11 wk. (D) Three-dimensional isosurface reconstructions of branch tips in (C). (E) High-magnification view of branch tip #15 (green box in [A and B]). (F) Three-dimensional isosurface reconstructions of branch tips in (E). (G) Plot of change in BTL of dynamic branch tips as a function of age. Number to the right denotes branch tip number. Scale bars: (A and B), 25 μm; (B–F), 5 μm.

We next examined layer 2/3 non-pyramidal neurons. Figure 3 shows maximum-intensity *z*-projections (MZPs) of image planes close (± 15 μm) to the cell body of a non-pyramidal neuron “nmr” with a bitufted dendritic morphology revealing its highly complex local arborization (Figure 3A and Video S2). The cell body's center of mass was 118 μm below the pial surface. This neuron had four primary dendrites with a total of 49 branch tips. Twenty-eight of the 49 branch tips were monitored for 4 wk. Four of the 28 branch tips exhibited variations in length. Two examples are shown where branch tips visibly elongated in the *x–y* plane (Figure 3A–F). Branch tip #20 elongated by approximately 16 μm over 4 wk (Figure 3C, 3D, and 3G). Concurrently, branch tip #15 increased in length by approximately 10 μm (Figure 3E–3G). Both branch tips emanate from the same primary dendrite whose dendritic branch length accounts for 62% of the total monitored dendritic length of the neuron. These results demonstrate that dendritic arbors of neurons within the adult neocortex are capable of growth.

**Figure 4:**
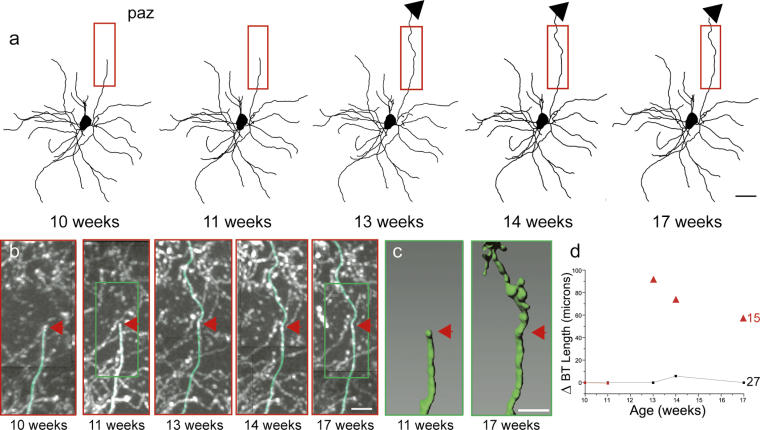
Large-Scale Dendritic Branch Growth in a Non-Pyramidal Neuron (A) Three-dimensional skeletal reconstructions of the non-pyramidal neuron “paz” from images acquired over 7 wk. Note that a growing branch tip exceeded the imaging volume after 11 wk, and black arrowheads denote its postulated continuation. (B) MZPs of region outlined by red box in (A). The branch tip (#15) elongates radially in the *x–y*-axis and away from the pial surface in the *z*-axis. Non-specific labeling in the MZP is exacerbated by the summation of additional *z* stacks due to elongation on the *z*-axis. The traced branch of interest is highlighted by a green overlay. Red arrowheads mark the approximate distal end of the branch tip at 11 wk. (C) Three-dimensional isosurface reconstructions of the region of the branch tip outlined by a green box in (B). (D) Plot of change in BTL of dynamic branch tips as a function of age. Triangles denote the minimum length of the branch tip as it exceeds the border of the imaging volume. Number to the right denotes branch tip number. Scale bars: (A), 25 μm; (B and C), 10 μm.

A different non-pyramidal neuron, “paz,” residing 78 μm below the pial surface, is shown in Figure 4. Two-dimensional projections of the three-dimensional traces show a moderately branched interneuron with a bitufted dendritic morphology (Figure 4A). This cell had seven primary dendrites with 47 branch tips. Twenty-nine of the 47 dendritic branch tips were monitored for 7 wk. Virtually all the branches were stable. However, two branches exhibited remodeling, one of which was so large in scale that it exceeded the imaging volume. Time-lapse images revealed that within as little as 2 wk, this branch tip more than doubled its length and exited the imaging volume (Figure 4). Although at 13 wk postnatal we were unable to follow the process to its termination, we measured a net extension of >92 μm from the branch tip at 11 wk to its location at the edge of the imaging volume 2 wk later. The axon of this neuron was found to project from the cell body in the opposite direction of this changing dendritic branch tip. This dramatic increase in BTL indicates that neurons in adult visual cortex have the capacity for large-scale remodeling.

**Figure 5:**
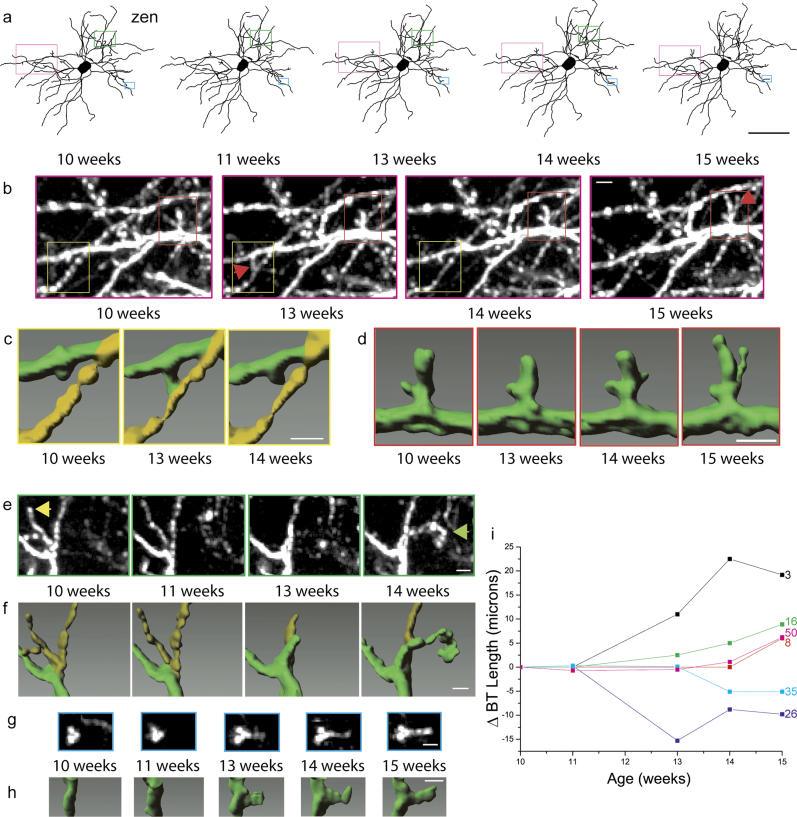
Branch Extensions, Retractions, and De Novo Branch Tip Addition in a Non-Pyramidal Neuron (A) Three-dimensional skeletal reconstructions of the sparsely spinous non-pyramidal neuron “zen” from images acquired over 5 wk. (B) High-magnification MZP view of region outlined by purple box in (A). Red arrowheads indicate examples of structural remodeling. Three-dimensional isosurface reconstructions show (C), the elongation and retraction of a spine toward an axon (yellow overlay). (D) Structural change in a cluster of branch tips (#50, far right) (red box in [B]). (E) Higher-magnification MZP view of region outlined by green box in (A). Examples of process retraction and branch-tip (#3) addition are labeled with yellow and green arrowheads, respectively. (F) Three-dimensional isosurface reconstructions of (E) with axon in yellow overlay. (G) MZP view of region outlined by cyan box in (A) shows branch-tip (#16) addition on a different dendrite. (H) Three-dimensional isosurface reconstructions of (G). (I) Plot of change in BTL of dynamic branch tips as a function of age. Number to the right denotes branch tip number. Scale bars: (A), 50 μm; (B–H), 5 μm.

Although many non-pyramidal cells are spine-free, sparsely spinous non-pyramidal neurons were also observed and imaged. These cells typically exhibited multipolar dendritic morphologies (Figure 5A). The interneuron “zen” shown in Figure 5 had seven primary dendrites with 61 dendritic branch tips and seven spines on 2,814 μm (averaged over 5 wk) of monitored dendrite. The cell body's center of mass was 100 μm below the pial surface. The few spines on this neuron exhibited motility (Figure 5B and 5C) as previously described for spines on pyramidal neurons [10–13]. Six branch tips showed changes in length (Figure 5I). For example, branch tip #50 shown in Figure 5B and 5D elongated by approximately 7 μm (Figure 5I). In this cell we also observed a couple of rare de novo branch-tip additions. The initial addition of one new branch was first seen at 13 wk postnatal and could be observed elongating to approximately 22 μm by 14 wk postnatal (Figure 5E, 5F, and 5I). Also in the proximity of the branch addition, we observed the retraction of a putative axon of unknown origin (Figure 5E and 5F). A second branch-tip addition was concurrently seen on an independent dendrite (Figure 5G and 5H), extending approximately 9 μm from 11 wk to 15 wk postnatal (Figure 5I).

Every one of the non-pyramidal cells imaged from layer 2/3 showed at least one and as many as seven changing dendritic branch tips. On average, approximately 14% of the monitored branch tips on non-pyramidal interneurons showed structural rearrangement. Of these, 3% were new branch-tip additions, 2% were loss of existing branch tips, and the rest were approximately half elongations and half retractions. Remodeling in some cases was incremental but could also occur in short temporal bursts. Changes were never observed in primary, first-order, dendritic branches, but they were otherwise not restricted by branch order. These data demonstrate that even without peripheral perturbations, branch tips of non-pyramidal cells in the superficial layers of the adult neocortex exhibit elongation, retraction, and branch-tip addition.

### Dynamically Remodeling Neurons Express GABA

**Figure 6:**
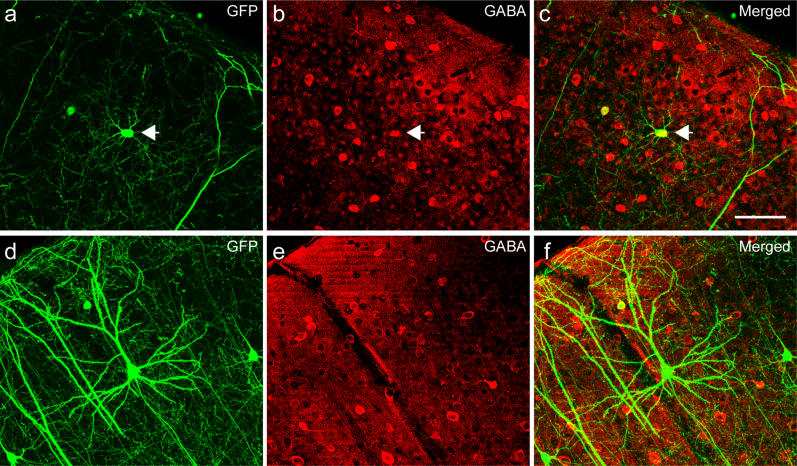
Remodeling Adult Non-Pyramidal Neurons in the Superficial Layers of the Visual Cortex Are Inhibitory GABAergic Interneurons (A) Confocal image stack of a coronal section containing the chronically imaged non-pyramidal neuron “ttr” is visualized by GFP staining (green, filled arrows) and is immunopositive for GABA (visualized in red) (B), overlay of GFP and GABA shown in (C). (D) Confocal image stack of a coronal section containing the chronically imaged pyramidal neuron “dow” is immunonegative for GABA (visualized in red) (E), overlay of GFP and GABA shown in (F). Scale bar: (A–F), 100 μm.

Neocortical interneurons are a diverse population with distinct morphological, physiological, and molecular subtypes [19–23]. Three of the eight imaged non-pyramidal neurons were unequivocally identified in coronal sections after post-imaging immunohistochemistry based on morphology and location. All of these cells were immunopositive for GABA (gamma-aminobutyric acid) (Figure 6A–6C), while pyramidal cells were GABA-negative (Figure 6D and 6F).

DAPI staining located the border between layer 1 and layer 2/3 at ∼80 μm below the pial surface (Figure S1A–S1D), consistent with previous findings [24], thus placing the imaged non-pyramidal cell bodies within layer 2/3 or at the layer 1–2/3 border (Figure S1). In an attempt to further classify their subtype, we also probed the sections for parvalbumin, somatostatin, and cholecystokinin, but found the imaged neurons negative for all three (WCAL and EN, unpublished data). This was not surprising given the low representation of these subtypes in layers 1 and 2/3 of the visual cortex in both GFP- and non-GFP–labeled GABA-positive interneurons (Figure S1E). From 158 GFP-positive non-pyramidal cells in seven animals, 92% (SEM = 2.1%) were GABA-positive. Since virtually all the non-pyramidal interneurons in the superficial layers of the visual cortex are GABA-positive, it is likely that the five imaged neurons that were not successfully identified in the sectioned brains were also GABAergic, strongly suggesting that dendritic arbor remodeling in adult neurons occurs predominantly in inhibitory GABAergic interneurons.

## Discussion

Although the capacity for change in the adult brain is limited compared with development, the adult cerebral cortex does maintain a degree of plasticity (reviewed in [4]). Electrophysiological recordings and anatomical analysis both suggest that potential sites for this plasticity are the horizontal connections within the superficial cortical layers [7,25–27]. Additional evidence comes from molecular studies demonstrating that in the superficial layers of adult striate cortex, visual input transcriptionally regulates genes involved in process outgrowth [28–31]. Together these data led us to hypothesize that neurons in the superficial layers of the adult cortex can undergo arbor remodeling without extreme peripheral perturbation. To test this hypothesis, we imaged the dendritic arbors of neurons in the visual cortex of adult mice over several months. Our findings show that in layer 2/3 the dendritic structure of pyramidal neurons is stable, while inhibitory interneurons undergo dendritic arbor remodeling.

Included in the analysis were 62% of the non-pyramidal dendrites, while 42% of pyramidal dendrites were successfully monitored. Given that 35 of the 259 monitored non-pyramidal branch tips changed, if the two cell types were equivalently dynamic, then the probability of a branch tip change event can be estimated as 35/259 = 0.135. In 124 monitored pyramidal branch tips, we would expect to have observed at least one branch tip change of this type with probability 1–10^−8^ (Protocol S1). Therefore, the probability that we missed an event due to sampling issues is 10^−8^. The fact that we did not observe any changes in the pyramidal branch-tip group allows us to reject the null hypothesis that the change probabilities for the two groups are the same, in favor of the more plausible alternative that the two groups have significantly different dynamic properties. Arguing against the possibility that missed events could be accounted for by a sampling bias is the fact that when comparing the size distributions of sampled branch tips for the pyramidal and non-pyramidal population, there is a similar sampling of processes in the 40- to 120-μm range (Figure S2), the size range where most changes occur within the non-pyramidal population (Figure S3).

Although there are excellent studies in the field that promote the view that adult neocortical structure is stable, to date all of them focus on pyramidal cell morphology, and most studies focus on spine dynamics [11–13]. Our data do not contradict but rather complement these studies. While our results are consistent with previous reports on stability of the apical dendrites on layer 5 pyramidal cells [10], we do not exclude the possibility that pyramidal neurons undergo any arbor remodeling, especially in response to perturbations of the sensory periphery [32]. Rather, we suggest that under normal conditions their structural rearrangements are less pronounced than those seen in layer 2/3 interneurons.

In felines and primates, the physiological plasticity manifested by cortical cells during the critical period for development of eye-specific preference is accompanied by clear activity-driven segregation of geniculocortical afferents into ocular dominance columns. Yet, there are many instances where physiological plasticity can be seen in the superficial cortical layers of adult animals without such a clear anatomical “readout” [33–35]. The scale of structural change during circuit refinement in the adult may be below the threshold for visualization with the anatomical methods used to monitor layer 4 afferent segregation. It is understandable that the structural remodeling described here was previously undetected by classical anatomical methods relying on sample statistics, as the changes are on the order of tens of microns (small relative to the entire dendritic arbor); occur on a subset of dendritic branches in a subset of cell types; and can occur sporadically in bursts of remodeling. In addition, when dendritic branches of the same neuron both grow and retract, the total net change may be negligible (∼1%–5% of the total monitored dendritic branch length). A technology where the same neurons can be vitally imaged over days and weeks in the intact brain allows for discrimination of small-scale structural dynamics, opening to reinterpretation the apparent disconnect between functional plasticity measured electrophysiologically in the extragranular layers and the absence of measurable anatomical change. Our results using chronic in vivo imaging demonstrate that there is an intrinsic capacity for structural remodeling in the superficial layers of the adult neocortex. Unlike during development when changes in dendritic arbors are widespread, changes in the adult are localized to a small subset of processes. The change in these individual processes on the scale of 20–90 μm, however, may be large enough to influence receptive field properties. The changing tips are specific to a certain neuronal subtype, suggesting cell type–specific rules to this remodeling. It has yet to be shown that this minority of “plastic” dendritic processes are ones that underlie the functional reorganization of adult cortical maps measured electrophysiologically. As there is previous evidence for horizontal axonal sprouting in the adult striate cortex following retinal lesions [7], and in the somatosensory cortex following whisker trimming [36], another area for further investigation would be the structural plasticity of axonal arbors in the long-range pyramidal cells of layer 2/3, and how they relate to dendritic changes.

**Figure 7:**
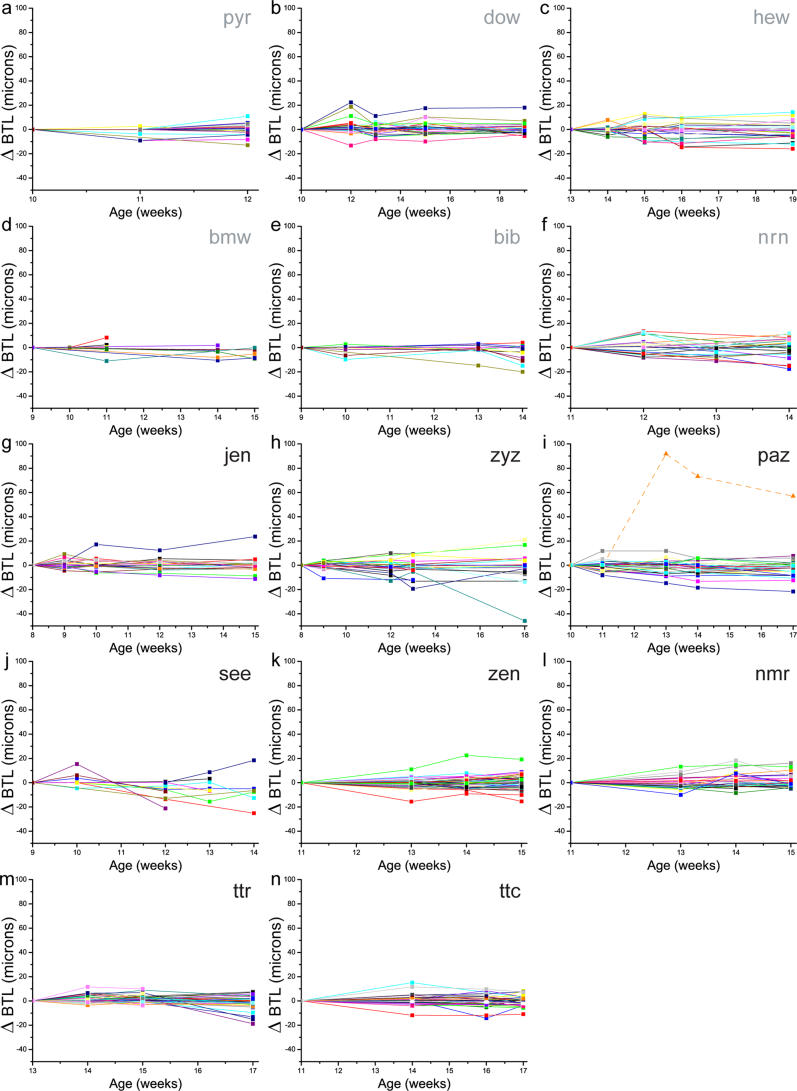
The Change in BTL is Plotted for Each Individual Monitored Branch Tip of Every Imaged Cell Three-letter code, top right. (A–F) pyramidal cells; (G–N) non-pyramidal cells. Triangles and dashed lines denote the minimum length of the branch tip as it exceeds the border of the imaging volume.

Unresolved questions in the field are whether and what kind of structural changes are expected in adult plasticity and how best to detect and analyze them. If averaged across the entire arbor, the changes in BTL of non-pyramidal neurons correspond to approximately 5% of the monitored dendritic branch length. However, individual branch tips changed by 16% to 456% (not including new branch-tip additions), with average elongations and retractions of 16 μm. Net changes ranged from −5% to +8% of the average monitored branch length. Since changes are localized, it may be preferable for both detection and analysis methods to be implemented on a process-by-process basis rather than by conventional approaches that rely on statistical averaging across all the processes of a cell or an entire cell population. In conventional analyses, averaging across the entire arbor would bias against events with potential functional importance if they occurred in a minority of dendrites. Even when viewed on a process-by-process basis, clearly changing branch tips can be difficult to identify when looking at the absolute change in BTL (Figure 7). For example, branches on cells such as “nmr” and “zen” (shown in Figures 3 and 5, respectively) that are unambiguously changing may be scored as false negatives. Thus, an alternative analysis that better represents the data scored by direct observation is clearly needed. We found that scaling the changes in length by the average length of the dendritic branch tip enhanced our ability to detect changes and represented salient changes in structure better than a non-scaled analysis. Advances in such quantitative analysis of morphometric measures over time will determine how to best represent structural change of neurons in the adult cortex.

Twenty percent to 30% of the neurons in the neocortex are non-pyramidal interneurons, and most adult neocortical interneurons are considered inhibitory, using GABA as a neurotransmitter [19–23]. A diverse population of non-pyramidal interneurons preferentially populate the superficial layers of the neocortex and are characterized by their morphological, electrophysiological, molecular, and targeting properties [21,23]. Most mature non-pyramidal interneurons lack dendritic spines, and most project locally, usually arborizing within a cortical column or projecting horizontally across columns, but rarely projecting to distant brain regions [37]. Inhibitory interneurons are thought to have an important role in modulating excitatory circuitry by depressing, blocking, or sculpting the temporal response properties of excitatory neurons [38,39]. During development, inhibitory circuitry is crucial for the onset of critical-period ocular-dominance plasticity [40–43] (reviewed in [44]), plasticity of the somatosensory cortex [45], and refinement of visual receptive fields [46,47]. By shortening stimulus-evoked spike trains in immature neurons, GABAergic activity can decrease the temporal asynchrony of uncorrelated inputs [48]. In addition, interneurons can coordinately synapse onto nearby excitatory pyramidal cells in a developing network, locally synchronizing their spike timing. Both shortening prolonged discharge and orchestrating spike timing could enhance the ability of target neurons to participate in spike timing–dependent plasticity [45,46]. Interestingly, in adult monkeys monocular deprivation modifies the expression of GABA and glutamic acid decarboxylase in the primary visual cortex in an eye-specific manner [49], suggesting that GABAergic transmission is sensitive to activity-dependent plasticity in the adult. Our data indicating that the structural plasticity of interneurons is continuous through adulthood raises the intriguing possibility that local remodeling of inhibitory connections may underlie adult cortical plasticity. This finding would have important implications for models of cortical functional circuitry and its activity-dependent modulation.

## Materials and Methods

### Animal surgery

*thy1*-GFP-S mice [17] were anesthetized with 2.5% Avertin (0.015 ml/g IP), and anaesthesia was monitored by breathing rate and foot-pinch reflex. The skull overlying both visual cortices [24] was carefully removed, leaving behind the dura, and 5-mm-diameter circular glass cover slips (No. 1) were positioned over the openings and sealed in place with Palacos R bone cement. Following surgery, mice were given lactated Ringers solution (0.015 ml/g SC) and Buprenix (0.3 mg/ml SC 2× daily for 5 d) as an analgesic and returned to individual cages for recovery under observation. Surgeries were performed at 4–6 wk postnatal to allow at least a 2- wk recovery before imaging.

### In vivo two-photon imaging

In vivo two-photon imaging was achieved using a custom-built microscope and acquisition software [18] modified for in vivo imaging by including a custom-made stereotaxic restraint affixed to a stage insert for the motorized stage (Prior Scientific, Cambridge, United Kingdom). While designed to run at high acquisition rates, for these experiments a conventional scanning rate was used to increase signal intensity by locking the polygonal mirror and using both raster-scanning mirrors. The light source for two-photon excitation was a commercial Ti:Sapphire laser, Mira (Coherent, Santa Clara, California, United States), pumped by a 10-W solid-state laser delivering 150 fs pulses at a rate of 80 MHz with the power delivered to the objective (with a transmittance of 20% to 30%) ranging from ∼100–250 mW depending on imaging depth. The excitation wavelength was set to ∼890 nm, with the excitation signal passing through an Achroplan 40×/0.8 NA water-immersion objective (Zeiss, Oberkochen, Germany) and collected after a barrier filter by a photomultiplier tube. Due in part to the sparse labeling of cells in the superficial layers of the thy1-GFP-S neocortex, the same cells could be identified and re-imaged for up to 3 mo using local fiduciary landmarks of the brain's surface vasculature.

### Image acquisition and analysis

Adult mice (8–19 wk postnatal) previously implanted with cranial windows were anesthetized with 2.5% Avertin (0.015 ml/g IP). Anaesthesia was monitored by breathing rate and foot-pinch reflex, and additional doses of anaesthetic were administered during the imaging session as needed. The head was positioned in a custom-made stereotaxic restraint affixed to a stage insert for a motorized stage (Prior Scientific). Nine slightly overlapping volumes in a 3 × 3 array were imaged through *z–x–y* translation of a motorized stage (*z* spacing ∼1.5 μm). Due to variations in head position across imaging sessions, cells of interest were not always centered in the imaging volume. These shifts in registration slightly affected image borders, so that a fraction of dendritic branch tips were excluded at any given imaging session. Since the direction of shift is random, there was no intentional bias in exclusion of tips for a particular neuron or for a particular imaging session and the same exclusion rules applied to all neurons. However, the longer process radius of the pyramidal cell dendrites potentially biased against their sampling (see the Discussion section regarding this point). Raw scanner data were processed in Matlab (Mathworks, Natick, Massachusetts, United States) and ImageJ (National Institutes of Health, Bethesda, Maryland, United States). Individual image planes were stitched together (VIAS version 2.1, http://www.mssm.edu/cnic/tools.vias.html) such that each is a 3 × 3 montage of adjoining *x–y* sections at a given depth from the pial surface. Four-dimensional *(x, y, z, t)* stacks were traced and analyzed blind to age using Object-Image (http://simon.bio.uva.nl/object-image.html) [50] and Neurolucida (MicroBrightField, Williston, Vermont, United States). Three-dimensional surface reconstructions were generated using Imaris (Bitplane AG, Zurich, Switzerland).

The analysis included 124 branch tips from six pyramidal cells in six animals and 259 branch tips from eight non-pyramidal cells in seven animals at least 65 μm from the pial surface (two non-pyramidal cells, “ttr” and “ttc,” were from the same animal) ranging in age (at the time of imaging) 8–19 wk postnatal (Figure 1). Cells were arbitrarily named with a three-letter code. BTL was measured as the linear arc length from well-defined distal ends to the first encountered branch point. Axons were not included in the skeletal tracings. Dendritic and axonal branches were distinguished by morphology. Axons were typified as tubular, thin (sometimes less than the point spread function of the microscope) processes, often studded with varicosities every few microns. Dendrites were distinguished by thicker diameters (generally >2 μm), smooth, gradually tapering processes, and characteristic branching patterns. Since we could not measure the same branch tip multiple times for a given time point, we used the time-lapse measures of the pyramidal and non-pyramidal branch tips that did not show change as an upper bound of the measurement error we would have observed if we had made multiple measurements at the same time. The average SEM was 1.9 (standard deviation = 1.4) μm for the monitored pyramidal cell branch tips and 1.1 (standard deviation = 0.8) μm for non-changing non-pyramidal branch tips. The larger measurement error in pyramidal branch tips can be attributed to the extension of long branch tips over multiple stitching boundaries.

### Immunohistochemistry

Previously imaged mice were heavily anesthetized with 2.5% Avertin (0.030 ml/g IP) and their brains processed for immunohistochemistry essentially as described [51]. Sections were first incubated with GABA (rabbit polyclonal antibody; 1:5000; Sigma, St. Louis, Missouri, United States), followed with Alexa555 conjugated goat IgG secondary antibodies (1:400; Molecular Probes, Eugene, Oregon, United States). Alternatively, sections were first incubated with parvalbumin (monoclonal antibody; 1:1000; Sigma) and somatostatin (rabbit polyclonal antibody; 1:1000; Chemicon, Temecula, California, United States) followed with appropriate Alexa555 and 647-conjugated goat IgG secondary antibodies (1:400; Molecular Probes). After visualization, sections were unmounted in PBS and reprocessed using cholecystokinin (monoclonal antibody #9303; 1:1000; CURE/Digestive Disease Research Center, VAGLAHS, Los Angeles, California, United States) and GABA (rabbit polyclonal antibody; 1:500; Chemicon), followed with appropriate secondary antibodies. Imaged cells were identified by location, morphology, and local landmarks. Images were acquired with a Fluoview confocal (Olympus, Tokyo, Japan) or an upright epi-fluorescence scope (Nikon, Tokyo, Japan) using a 20×/N.A. 0.5 (Olympus), 20×/N.A. 0.75 (Nikon), or 40×/N.A.1.30 (Nikon) objective.

## Supporting Information

Figure S1 Layer Localization of Imaged Neurons and Distribution of GABAergic Subtypes (A and C) Neurons shown in Figure 6 were in layer 2/3 as shown by DAPI staining (B and D) (border of layer 1 and layer 2/3 delineated by empty arrowheads). (E) GFP labeling of non-pyramidal cells in layers 1 and 2/3 shows a representative distribution of GABAergic, parvalbumin, somatostatin, and cholecystokinin immunopositive cells. Solid bars represent percent of layers 1/2/3 GFP-labeled non-pyramidal cells immunopositive for a given marker, and empty bars represent percent of all GABA-positive cells immunopositive for the same markers. Error bars represent SEM. Scale bar (A–D): 50 μm.
(5.8 MB TIF).

Figure S2 Histogram of the Number of Monitored Branch Tips for Different Branch Lengths of Pyramidal and Non-Pyramidal Cells (837 KB TIF).

Figure S3 Histogram of the Number of Remodeling Events in Non-Pyramidal Neurons as a Function of Distance from the Cell Soma (899 KB TIF).

Protocol S1 Analysis of the Dendritic Change Propensities for Non-pyramidal and Pyramidal Neurons (47 KB DOC).

Video S1 *Z*-Stack of Pyramidal Cell “dow” Descending at 1.5-μm Steps (1.4 MB WMV).

Video S2 *Z*-Stack of Non-Pyramidal Cell “nmr” Descending at 1.5-μm Steps (862 KB WMV).

## Abbreviations

BTL: branch tip length
GABA: gamma-aminobutyric acid
GFP: green fluorescent protein
MZP: maximum-intensity z-projection
