# Correction: Dynamic Remodeling of Dendritic Arbors in GABAergic Interneurons of Adult Visual Cortex

**DOI:** 10.1371/journal.pbio.0040126

**Published:** 2006-05-16

**Authors:** Wei-Chung Allen Lee, Hayden Huang, Guoping Feng, Joshua R Sanes, Emery N Brown, Peter T So, Elly Nedivi

## Abstract

Chronic in vivo imaging of fluorescent-labeled neurons in adult mice reveals extension and retraction of dendrites in GABAergic non-pyramidal interneurons of the cerebral cortex.

In *PLoS Biology*, volume 4, issue 2, DOI: 10.1371/journal.pbio.0040029

The authors wish to add to the Acknowledgments that Emery N. Brown's work on the project was supported in part by a grant from the National Institute on Drug Abuse of the National Institutes of Health.

