# Supplementary material for: Dynamic Remodeling of Dendritic Arbors in GABAergic Interneurons of Adult Visual Cortex
Source: PLoS Biol. 2005 Dec 27;4(2):e29. doi: 10.1371/journal.pbio.0040029 (PMC1318477; doi:10.1371/journal.pbio.0040029)
Supplement: Supplementary file 1 [file pbio.0040029.sd001.doc]

**Nedivi Supplementary Text**

**Analysis of the Dendritic Change Propensities for Non-pyramidal and Pyramidal Neurons**

How likely is it that we missed observing changing branch tips in the pyramidal neurons if at least one had occurred when we monitored 124 pyramidal neuron branch tips and we assume the probability of a change was equal to that estimated for the non-pyramidal neuron branch tips p = 35/259?

To answer this question, we assume that the propensity to change for branchtips of the pyramidal neurons is the same as for the branchtips of the non-pyramidal neurons. That is, our null hypothesis is that the two types of neurons have the same propensity to changes their dendritic branchtips. We want to calculate how likely is our observation of no branchtip changes observed among the pyramidal neuron branchtips if the two branchtip change probabilities are same. This is equivalent to asking how likely is it that we missed observing all the possible change events in branch points of the pyramidal neurons. Therefore, we estimate the probability of a pyramidal neuron changing from the non-pyramidal neurons as

$p=35/259=0.\mathop{135}\limits^{\_\_\_} .$ (S.1)

The desired probabilities in this problem can be computed from the binomial distribution which defines the probability of observing exactly $k$changing events for a given$N$, the number of monitored pyramidal branch tips, and a given$p$, the probability of a branchtip changing. This is given as (Ross, 1997)

$\Pr( of exactly k changes)=\left(\frac{N!}{k!(N-k)!}\right)p^{k}(1-p)^{N-k}$ (S.2)

where $k!=1\cdot2\cdot3...\cdot k.$ The question to now ask is, What is the probabililty that we should have seen at least one changing event for the pyramidal neuron branch tips if they had the same change probability $p$ as the non-pyramidal neurons? This is

$\begin{matrix} \Pr( of at least one change event)=\sum_{j=1}^{\infty} \Pr(of j changing events) \\ =1-\Pr(zero changing events). \end{matrix}$ (S.3)

Since we monitored 124 pyramidal branchtips then,

$\begin{matrix} \Pr( of at least one change event)=1-\Pr(zero changing events). \\ =1-\left[\left(\frac{N!}{N!0!}\right)\right]p^{0}(1-p)^{N-0} \\ =1-\left[\left(\frac{124!}{124!0!}\right)0.135^{0}(0.865)^{124}\right] \\ =1-\left[1\times1\times1\times10^{-8}\right] \\ \approx1 \end{matrix}$ (S.4)

This calculation tells us that if the propensity for the pyramidal branchtips to change were equal to the propensity of the non-pyramidal neurons to change, then the probability of seeing at least one change in the pyramidal branchtips would be one. Thus, we should have seen a pyramidal branchtip change event with near total certainty. Stated another way, the probability of missing this event is the probability of observing any other event except this one. Since this event is the event that at least one branchtip changed, the probability of not observing or missing this event is$\Pr(zero changing events)=10^{-8}$ (Eq. S.4).

The fact that we did not observe any change events in the pyramidal branchtip group allows us to reject the null hypothesis that the change probabilities for the two groups are the same in favor of the more plausible alternative that the two groups have significantly different dynamic properties.

We could not use the standard Poisson approximation to the binomial rare events computation in these analyses because the expected number of occurrences, i.e., 17 events, was too high to satisfy the conditions for this approximation to be accurate.

To be complete, we consider two other cases. First, if for the pyramidal neurons $p$>0.135, then $\Pr(zero changing events)<10^{-8}$. Second, if 0.05<$p$ < 0.135 $\Pr(zero changing events)<2\times10^{-3}.$ Hence, for a change probability as small as 0.05 the probability of observing no branchtip change events in a sample of 124 pyramidal branchtips is less than 0.002. Alternatively stated, the probability that you should have observed at least one of these change events is still, in this worse, case 0.998. This remains near certainty.

**REFERENCE**

Ross SM (1993) *Introduction to Probability Models*, London: Academic Press.
